# HIV testing amid COVID-19: community efforts to reach men who have sex with men in three Kenyan counties

**DOI:** 10.12688/gatesopenres.13152.2

**Published:** 2020-10-29

**Authors:** Manas Migot Odinga, Samuel Kuria, Oliver Muindi, Peter Mwakazi, Margret Njraini, Memory Melon, Bernadette Kombo, Shem Kaosa, Japtheth Kioko, Janet Musimbi, Helgar Musyoki, Parinita Bhattacharjee, Robert Lorway

**Affiliations:** 1 Men Against AIDS Youth Group, Kisumu, Kenya; 2 Mamboleo Peer Empowerment Group, Kiambu, Kenya; 3 HIV and AIDS People’s Alliance of Kenya, Mombasa, Kenya; 4 Partners for Health and Development in Africa, Nairobi, Kenya; 5 Ministry of Health, National AIDS and STI Control Programme, Nairobi, Kenya; 6 Institute of Global Public Health, University of Manitoba, Winnipeg, Manitoba, Canada

**Keywords:** COVID 19, Men who have sex with men, HIV testing, Kenya

## Abstract

In comparison to European and American countries, Kenya has been less impacted by the COVID-19 pandemic in terms of reported cases and mortalities. However, everyday life has been dramatically affected by highly restrictive government-imposed measures such as stay-at-home curfews, prohibitions on mobility across national and county boundaries, and strict policing, especially of the urban poor, which has culminated in violence. This open letter highlights the effects of these measures on how three community-based organizations (CBOs) deliver HIV programs and services to highly stigmatized communities of men who have sex with men living in the counties of Kisumu, Kiambu and Mombasa. In particular, emphasis is placed on how HIV testing programs, which are supported by systematic peer outreach, are being disrupted at a time when global policymakers call for expanded HIV testing and treatment targets among key populations. While COVID 19 measures have greatly undermined local efforts to deliver health services to members and strengthen existing HIV testing programs, each of the three CBOs has taken innovative steps to adapt to the restrictions and to the COVID-19 pandemic itself. Although HIV testing in clinical spaces among those who were once regular and occasional program attendees dropped off noticeably in the early months of the COVID-19 lockdown, the program eventually began to rebound as outreach approaches shifted to virtual platforms and strategies. Importantly and unexpectedly, HIV self-testing kits proved to fill a major gap in clinic-based HIV testing at a time of crisis.

## Introduction

On 11 ^th^ March 2020, the World Health Organization (WHO) declared the spread of COVID-19 a global pandemic after it swept across 114 countries, causing more than 4000 deaths. At the time of writing, SARS-CoV-2 has rapidly spread in 213 countries with an estimated number of 8.6 million persons being diagnosed with the virus, while claiming more than 460,000 lives globally ^1^.

In an effort to reduce the spread of the virus and mitigate the number of COVID-19-related mortalities, governments globally have introduced various public health measures, including restrictions on international and domestic travel and border controls; increased screening, testing and contact tracing; promotion and implementation of enhanced hygiene programs (i.e. regular handwashing and hand sanitizer use); and use of face masks and physical distancing in public spaces. Some countries have imposed partial or complete lockdowns and enforced curfews to regulate the movement of its citizens. Businesses have been forced either to operate from home or entirely shut down, rendering many workers, outside of those declared “essential”, unemployed. In economically disadvantaged countries like Kenya, these closures and travel restrictions are expected to have a dramatic impact on a national economy that derives most of its GDP from the tourism industry ^2^.

Although the COVID-19 epidemic has been less severe in East Africa compared to a number of Euro-American regions, since the announcement of the first COVID-19 case in Nairobi, Kenya, on March 12, 2020, the rate of infection across the country is projected to increase ^3^. At the time of writing, Kenya has reported over 37,000 COVID-19 cases across 34 counties, with 650 deaths related to the virus ^4^. The Kenyan government has enforced strict measures to prevent the spread of the corona virus and avert a health care crisis in an already fragile health system. These measures include a “dusk to dawn” curfew, inter-county mobility restrictions, mandatory use of face masks in public spaces, and forms of policing that have culminated in violence ^5^. Bearing the brunt are the urban poor and daily wage earners who have been intensely victimized by these policing measures ^6^.

Working on HIV programs with people whose behaviours are already criminalized in the Kenyan context, we are witnessing the unique and emergent struggles they are encountering ^7^. Kenya’s COVID-19 response measures have indeed imposed considerable obstacles to the effective delivery of HIV services to highly marginalized communities such as (SW), men who have sex with men (MSM) and people who inject drugs (PWID). In this paper, we specifically share the implementation experiences surrounding HIV prevention programs serving MSM in Kenya run by three community-led organisations: MAAYGO in Kisumu county, MPEG in Kiambu county and HAPA Kenya in Mombasa county. These programs have more recently attempted to strengthen HIV testing systems, a process that began prior to the COVID-19 epidemic. This program emphasis is highly motivated by recent global health HIV policies that compel program implementers to further their efforts in reaching the “undiagnosed” in order to achieve highly ambitious “90-90-90” (now “95-95-95”) population testing, treatment, and viral suppression targets ^8^. The programs that community organizations run to strengthen HIV testing in each of the three county sites in Kenya are technically supported through an ongoing collaboration with health scientists from the University of Manitoba; program specialists from the local NGO, Partners for Health and Development in Africa (PHDA); and health policy officials from The National AIDS and STI's Control Programme (NASCOP), Government of Kenya. In describing the impact of COVID-19 on the MSM community in three contrasting counties of Kenya, we specifically outline the strategies developed and implemented by the community-led organisations to ensure that HIV testing and other key prevention services remain available amid myriad COVID-19-related restrictions and the concomitant fears and anxieties of program beneficiaries.

## Impact of COVID-19 on MSM

The MSM-led organizations implementing HIV programs for MSM at each of the three sites mainly include working class men who, during COVID-19, have suffered job losses and unpaid leave like many other working class Kenyans. Many of these men have tended to gravitate toward particular forms of employment in what they regard as “safer” workspaces, but which have been extremely hard hit by the COVID-response measures: the hospitality industry, bars, barber shops and salons, and massage parlors. Many community members also depend on formal and informal modes of sex work, often to augment their income, and have seen the collapse of the sex industry with the closure of hotspots and other public spaces and venues where they normally gather to meet clients. Finding clients through virtual dating sites poses challenges for MSM for two reasons. For one, compared to female sex work, male sex work suffers the additional stigma of “homosexuality”, which has been criminalized by the Kenyan state, and thereby inhibits on-line advertising for sexual services for fear of blackmail. The Kenyan Penal Code and the Sexual Offences Act do not criminalise sex work, per se, but do criminalise the actions of third parties associated with sex work. However, Municipal by-laws across the country do directly criminalise sex work through articles outlawing “loitering for the purpose of prostitution,” “importuning” for the purpose of prostitution, and “indecent exposure.” ^i^. These laws impact MSM who do sex work. Second, efforts to adhere to physical distancing practices has greatly reduced the pool of available clients.

Loss of jobs and income has had dire financial consequences and led to food insecurity among MSM. Although receiving repeated requests for financial support from members, the ability of CBOs to respond to these needs more fully is greatly constrained by international funding systems on which their programs rely. Financial insecurity has forced some MSM to stay with friends to save on rent, thereby increasing the conditions of overcrowding and COVID-19 exposure risks ^9^. Being unable to afford rent, some MSM have been forced to vacate rented homes and now forced to stay on streets. With no source of income and limited resources to sustain livelihoods, it is virtually impossible for these men to afford personal protection equipment (PPE), as directed by the Kenyan government. A number of members who live with one of their sexual partners have begun to report experiences of intimate partner violence, including incidents of forced evictions. For those characterized as “noncompliant” with the government-related COVID-19 guidelines, especially violent, punitive actions taken by police in the form of beatings and arrest, have been brutal in their focus on sex workers, MSM and people who inject drugs. The general sentiment among members we have spoken to is one of anxiety, stress and hopelessness with regards to an unpredictable future.

## Impact on HIV testing

According to Kenya’s national guidelines, MSM, like other highly HIV vulnerable “key populations”, are expected to undergo regular testing every three months ^10^. This health service utilization strategy relies upon regular and intensive peer outreach work (to generate and maintain demand for health services), which must continually confront and break down access barriers related to pervasive stigma ^11^. Amid the COVID-19 pandemic, HIV testing has been greatly disrupted, including among those who regularly test, with clinical attendance waning considerably. Prior to COVID-19, the three CBOs had planned several activities for the period of February to April, 2020, which aimed to increase awareness and acceptance of, and demand for, HIV testing through the promotion and distribution of OraQuick ^®^ HIV self-testing (HIVST) kits. However, because most of these activities relied upon face-to-face outreach and interactions between peers, many of these activities were initially halted with the announcement of COVID-19 response measures. Previously, outreach workers regularly met with their peers in entertainment hotspots and other public venues and cruising places. However, regular and systematic peer outreach, which supported both regular testing in CBO-run clinics and the distribution of HIVST kits to peers, soon collapsed. Furthermore, CBO members were often fearful of coming to the clinic and interacting with clinical staff in each of the three sites, as rumours of COVID-19 transmission stirred fears in their communities.

**Figure 1.**
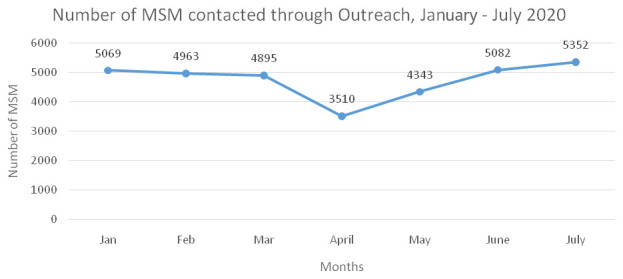
MSM contacted through peer outreach in the three sites (combined program data, Jan-July 2020).

**Figure 2.**
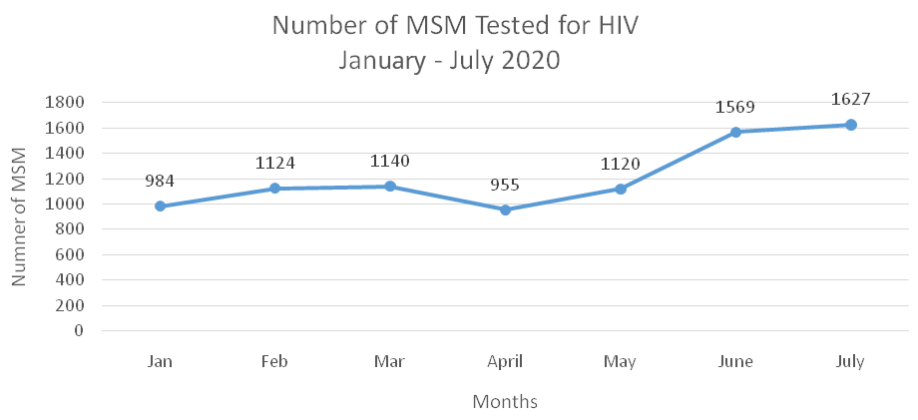
MSM testing for HIV in the three sites (combined program data, Jan-July 2020).

**Figure 3.**
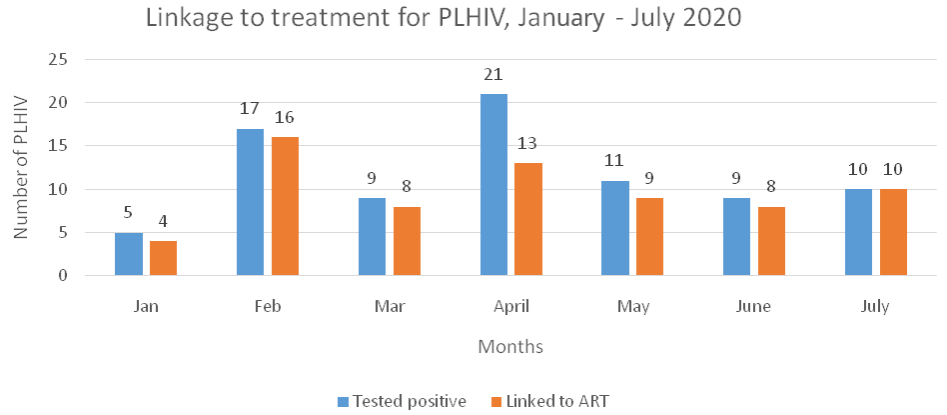
HIV treatment linkage among MSM who are living with HIV, in three sites (combined programme data, Jan – July 2020).

Physical distancing measures also initially forced the organizations to halt staff meetings, community health forums, and health promotion sessions. Evening curfews were imposed through “cessation of movement” orders within municipal districts, thereby disrupting delivery of services within each of the three sites. Fear and anxiety related to transmission of a new virus (i.e., SARS-CoV-2) also kept the community members away from the clinics. The MSM community could no longer readily move between their homes and the clinic to access services; nor could peer educators and service providers actually reach the clinical facilities (testing and linkage to treatment). Reviewing the combine program data from the three organizations from January to July 2020 shows how the restrictions impacted upon outreach and clinical services in April 2020 (see Figure 1, Figure 2 and Figure 3).

Although we see noticeable declines in the program performance indicators (peer outreach, HIV testing and linkage to ART) in the month of April 2020, we can also note some improvement in program performance since the month of May, which is owed to the innovative approaches that the community-based organizations (CBOs) undertook in each of the sites. 

## Adapting HIV testing programs in response to COVID-19

### Addressing safety concerns of service providers and program beneficiaries

**Figure 4.**
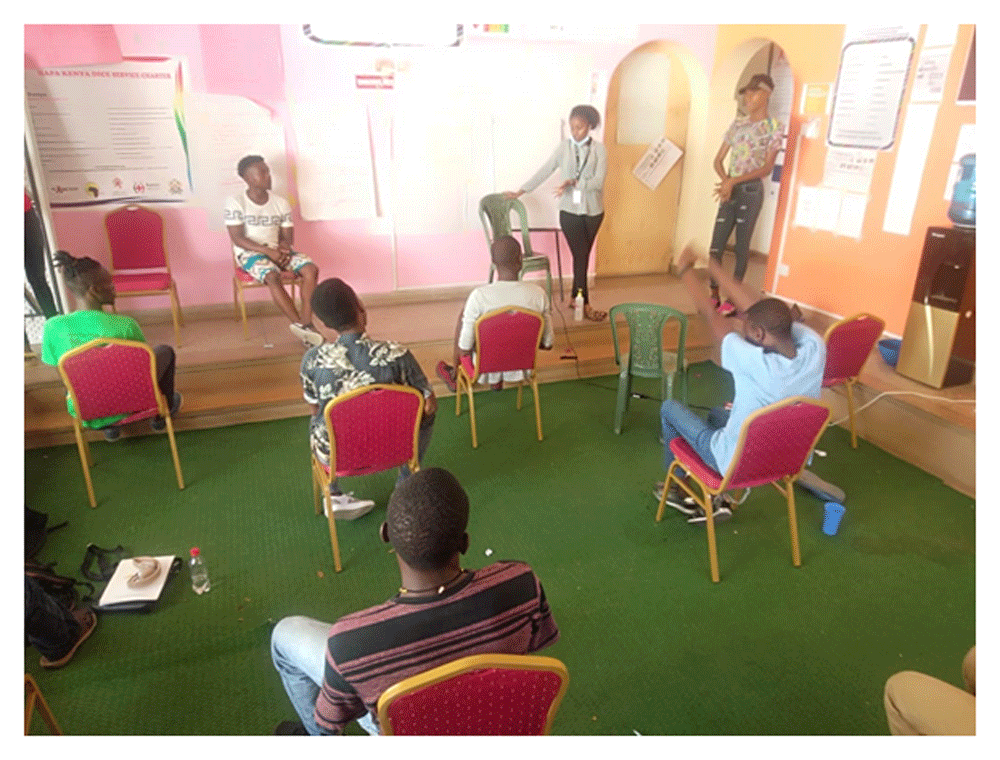
Intensive COVID-19 training conducted in HAPA Kenya offices, Mombasa.

**Figure 5.**
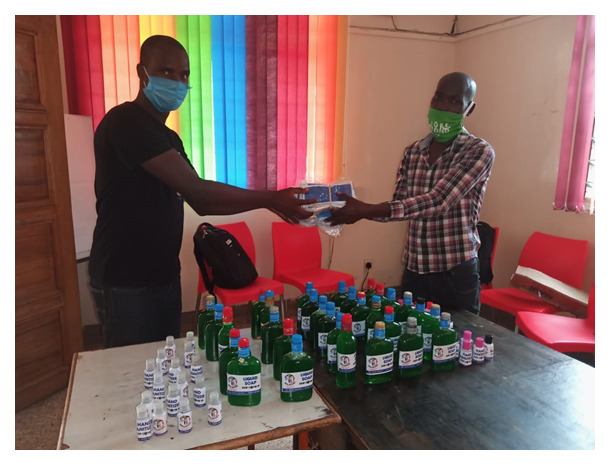
MAAYGO distributing hand sanitizers, face masks, and liquid soap to a partner organization.

**Figure 6.**
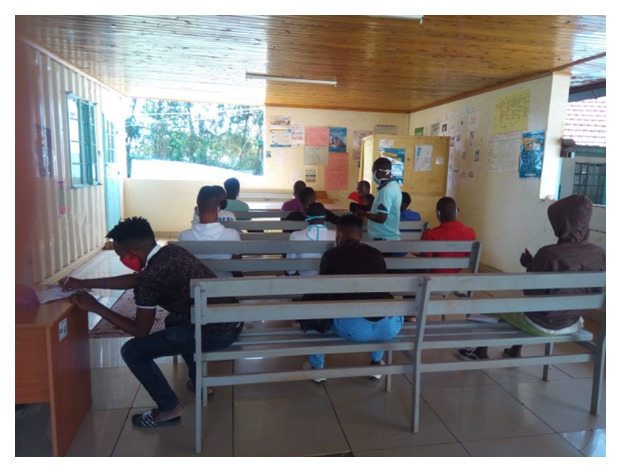
Clients maintaining physical distancing in the public health facility in Kiambu County.

Adhering to public health recommendations on COVID-19 infection control, the three CBOs trained their staff on COVID 19 transmission and prevention (see Figure 4). Moreover, the CBOs implemented various safety measures for their staff and program beneficiaries. Each organization placed a water dispenser and handwashing station at the entrance of the office and the clinic. Every individual accessing the premises therefore was expected to wash their hands as they entered. Although few international funders permitted the reallocation of HIV financial aid, UHAI-EASHRI ^ii^, Africa’s first indigenous activist fund supporting sexual and gender minorities and sex worker human rights, provided grants to respond to the COVID-19 pandemic, thus enabling CBOs to afford protective gear like gloves and masks for clinical staff and peer health workers. For community members and partner organizations unable to afford PPE, CBOs shared their supply of face masks and small bottles of sanitizers, which peer educators packed for distribution (see Figure 5). However, to adhere to physical distancing guidelines, the “safe space” of the three CBO drop-in centres were temporarily closed. Furthermore, the clinic reduced its daily hours of operation to three days a week in the month of April, while requiring clients to make advance bookings to keep the number of CBO clinic attendees to a minimum. While in the clinic, seating arrangements were clearly demarcated, 1.5 meters apart (see Figure 6). All members who visited the clinic were tested for an elevated temperature upon entrance.

In Mombasa, HAPA Kenya decided to formulate a standard operating procedure (SOPs) in response to COVID 19, with its main purpose of ensuring that all beneficiaries would have continued access to HIV prevention commodities. The SOP details how the commodities such as HIV self-test kits, condoms and lubricants, which were previously made available in hotspots, could be safely provided by the program. The SOP was designed to streamline operations for commodities in a way that protected both the CBO frontline health workers and the beneficiaries receiving the services. The SOP also provides programmatic guidance for the effective delivery of HIV commodities to the MSM community that carefully accounted for COVID-19 safety measures. For the SOP to be put into practice, the outreach, clinical and support staff were taken through an intensive training and sensitization sessions on COVID 19.

### Initiating and strengthening virtual outreach

**Figure 7.**
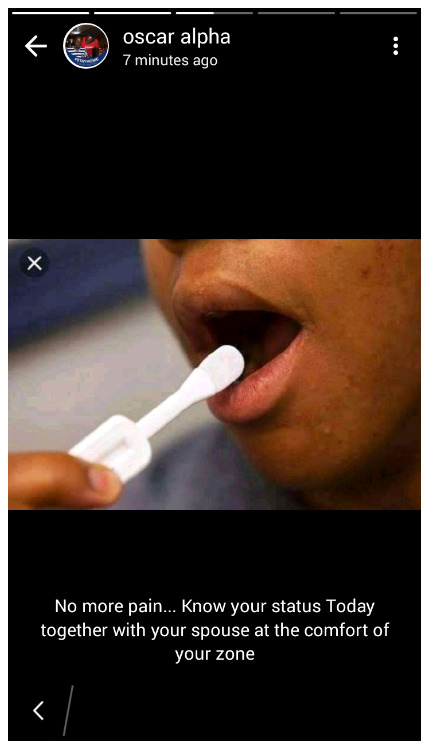
A screenshot showing one of the peer educators using WhatsApp platform to create demand for HIVST (The name “Oscar alpha” is a pseudonym).

In light of the shut-down of face-to-face peer outreach activities in public space, the programs soon shifted greater attention to their virtual outreach program to increase the utilization of social media platforms such as WhatsApp and Facebook. In Kiambu, MPEG scaled up their presence in the STEP 1 platform (a virtual platform to access confidential HIV and STI services) ^12^ through which they started making contact with new community members. MPEG sent more than 9000 messages related to COVID 19 and HIV testing to their members using the bulk SMS platform called the *Ujumbe SMS* ^13^. In Kisumu, each MAAYGO peer educator created a WhatsApp group for their peers where they could lead health education discussions on the spread of the new coronavirus and provide information on how one could access HIV prevention commodities and services during COVID-19. They used the group to also check-in on each other at a time when physical distancing frequently cut people off from vital social support networks. The WhatsApp groups were even used by some peer educators to reach new peers, enabling them to connect through existing social networks. In Mombasa, HAPA Kenya intensified their virtual outreach via dating sites such as Grindr and on Facebook pages by engaging new peer educators to conduct outreach in virtual spaces. Attempting to promote HIV and COVID-19 prevention, HAPA Kenya circulated messages across these virtual sites (see Figure 7) to address widespread fears among the MSM community and enhance trust and rebuild confidence in their organization. HAPA Kenya provided extra cellphone airtime to peer educators for communication, which was urgently needed to maintain contact with their extensive cohorts of contacts, through WhatsApp groups. As part of the health education work conducted on WhatsApp, peer educators increasingly began to notice and speak to emerging mental health issues such as stress, anxiety, fear of loss, while also reminding members to visit the clinic during appointed times to avoid overcrowding. The peers were also requested to encourage their friends to access available services at HAPA-Kenya’s drop-in center. The virtual team, comprised of 14 peer educators, made considerable effort to re-engage members who had once gathered in public hotspots, but had migrated to virtual spaces, so that they could be supplied with vital HIV services including HIVST kits. This team reported finding many of their lost contacts on new virtual platforms, such as TikTok (an app for sharing short videos).

### Delivering HIV testing services

**Figure 8.**
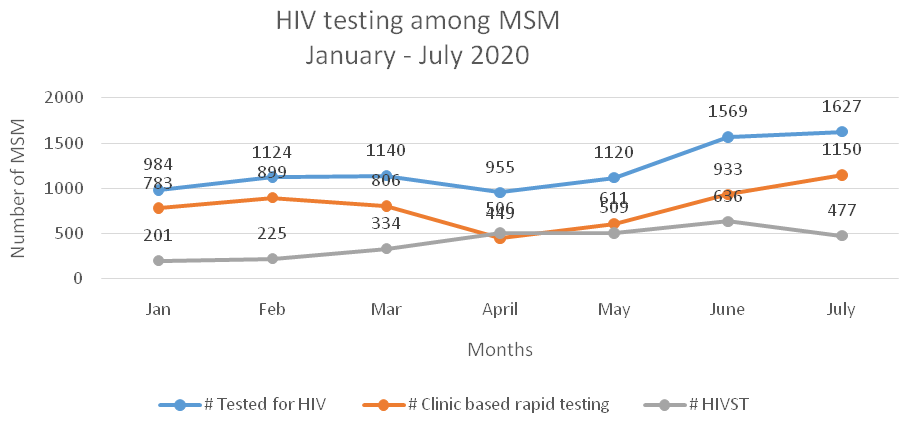
HIV testing among MSM, by type of test, in three sites (combined program data, Jan–July 2020).

In the COVID-19 era, CBOs employed innovative strategies to provide HIV testing services to their members. The organizations also saw an increase in demand for HIV Self testing kits. According to the program data, although HIV testing among MSM reduced in the month of April, the use of HIVST kits increased compared to clinic-based rapid testing (see Figure 8).

**Figure 9.**
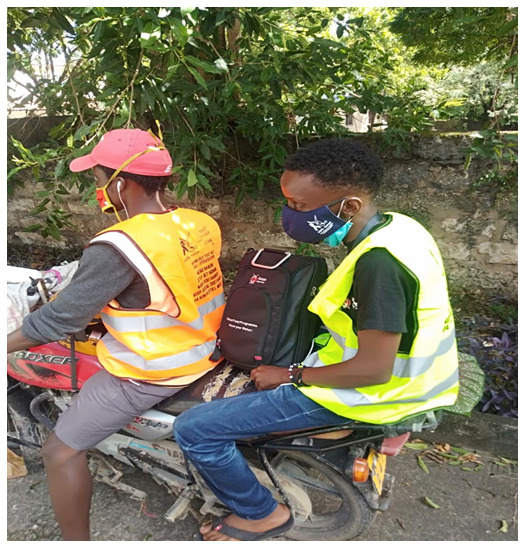
Peer educator from HAPA delivering commodities to clients on a motorbike.

In Kiambu, MPEG partnered with government health facilities, at the county level, to provide services to MSM. This translated into MPEG receiving support in terms of human resources and space. The latter form of support was vital for COVID-19 infection control as all government health facilities maintained strictly prescribed safety protocols. In Mombasa, HAPA Kenya employed a project van and hired public service motor bikes called *boda boda* to deliver condoms and HIVST kits (see Figure 9). Different boda boda riders were assigned to each of the sub-counties and their schedules were prepared in advance, on a weekly basis, by the outreach team to ensure timely delivery of commodities. In the context of Kisumu, when MAAYGO realised that clinic visits had begun to drop, they started to promote HIVST among those who (previously) frequently and occasionally tested for HIV at their premises. One of the goals of promoting HIVST was to ensure that MSM receiving kits were linked to further prevention and treatment services. During COVID-19, the program continued to implement a centralised follow-up system for those who reported having received and used HIVST kits. CBO clinicians and counsellors conducted follow-ups through phone calls with members.

### Ongoing communication and information sharing

**Figure 10.**
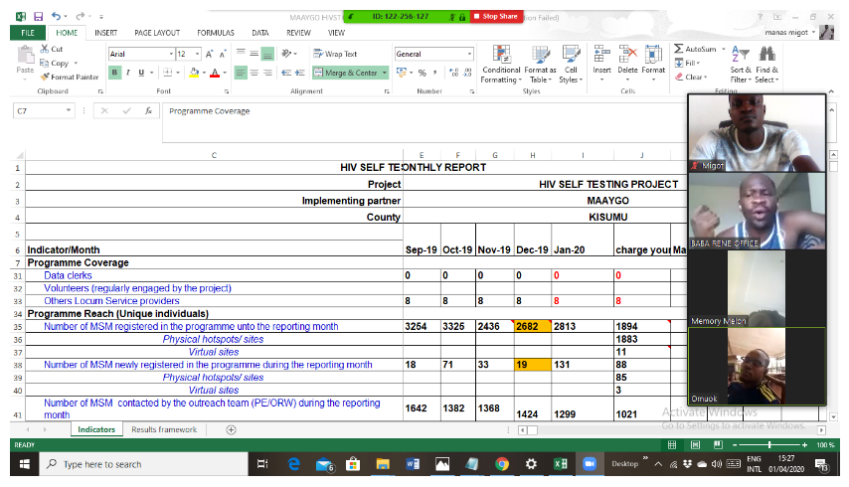
MAAYGO staff and outreach workers evaluating project implementation progress.

The MAAYGO team in Kisumu conducted virtual meetings with staff, peer educators and outreach workers, via zoom video communications (see Figure 10). One of the major and persistent challenges faced during these meeting, however, was internet instability, especially for team members located in rural areas.

In Mombasa, between May and June 2020, feedback and program updates between peer educators and managers were organized through group meetings with 15 staff members allowed at one time. In Kiambu, MPEG employed similar methods to conduct meetings with team members to keep abreast of program activities and emerging challenges. Staff and volunteers who needed to join these meetings were provided with extra airtime to enable their participation.

**Figure 11.**
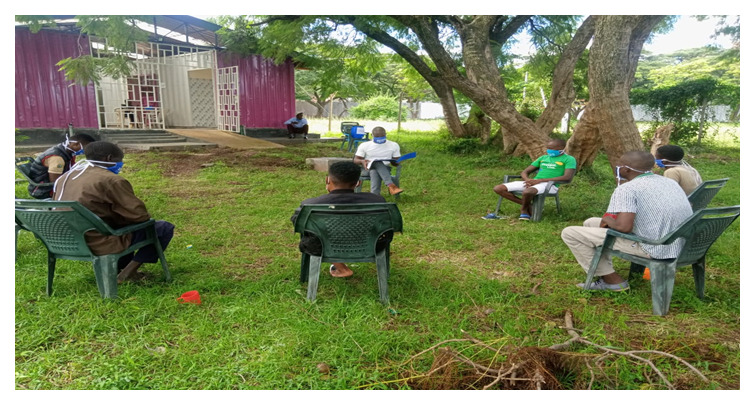
Meeting held between outreach workers and staff at Railways Health Centre, Kisumu.

More recently, the CBOs have begun to hold some face-to-face staff meetings, which take place under strict conditions: enough spacing between the meeting participants, wearing face masks, and frequent hand sanitization (see Figure 11).

### Other forms of support

In addition to disbursing HIV commodities and providing clinical support, the three CBOs provided other vital support in terms of nutrition and social benefits. All three CBOs applied for funding to organisations like UHAI EASHRI and Global Fund and generated additional resources through donations to purchase food packets that could be distributed to members of the MSM community, many of whom were experiencing food insecurity due to loss of income and employment. In some sites, the CBO staff arranged shelter for those who had become homeless. The organisations also worked with county governments to ensure that the poorest members of their community also received financial aid available through COVID-19-specific social protection programs initiated by the county.

### Flexibility in work plans and budgets

University of Manitoba and PHDA as technical partners, along with the CBOs, had to be flexible about work plans and budget as soon as the COVID-19 crisis struck. Emergent needs around COVID 19 were initially more immediate than HIV testing services, and so the budget was partially reallocated to purchase PPE for the staff and clients, as well as travel costs and airtime for staff to deliver health services on virtual platforms. The academic partners also cancelled some of the program monitoring and evaluation activities to enable the CBOs time to adapt to the new state of crisis. The guidance from NASCOP on providing services to key population during COVID 19 supported the CBOs to be flexible and innovative.

## Conclusion

Although, in terms of documented infections and mortalities, Kenya has been less impacted by the COVID-19 pandemic in comparison to other countries, our report of three CBOs struggling and persevering in their attempts to offer HIV services to highly stigmatized communities of MSM helps to illustrate the profound disruptions taking place in HIV program delivery [also see 14] in a part of the world where HIV continues to pose a major population health burden and challenge to the effective operation of health systems ^15^.

In the “end of AIDS” era, where aspirations of “elimination” run high in global health policy discourse that hinges “success” on the amplification of HIV testing and treatment targets ^16^, COVID-19 profoundly unsettles this optimism. However, despite the myriad challenges initially faced by the three Kenyan CBOs discussed in this report, especially in March and April 2020, towards the end of May program indicators showed a rebounding, greatly owed to the ingenuity and commitment of CBOs in their re-design of strategies for reaching their peers. We would like to recommend to community-led organisations and other implementers that in a crisis such as COVID they need to a) assess and understand the emergent needs of the community during such a crisis and respond to the most urgent needs; b) develop innovative strategies to reach the most vulnerable groups and provide services; c) address the fears and anxiety around the new pandemic among the implementing team and in the community and d) provide personal protective equipment to the staff and also, importantly, health insurance. 

These efforts of CBOs were supported by flexibility offered by some funders and the Ministry of Health, which provided guidance and policy support. The adaptation of outreach and program delivery and monitoring was also greatly supported through the flexibility offered by the technical partners; even though “strengthening HIV testing” and HIVST promotion was the initial goal of the collaboration, this flexibility accommodated the room to adapt to the emergent circumstances of COVID-19 with alternative ways of promoting HIV testing. Importantly, this flexibility afforded the space to reveal the unexpected utility of HIVST technologies in a time of crisis, helping community-based programs to resume their role in HIV service delivery amid restrictions on mobility. Indeed, the significance of the community-level responses—which shoulders the responsibility for delivering, distributing and promoting the use of vital HIV services and commodities—arguably must be more fully accounted for in any future epidemic or systemic crisis. Otherwise, we risk trading one crisis for the (re)emergence of another. We recommend to government, donors and technical partners to a) provide clear guidance and support during the pandemic; b) provide flexibility in relation to targets and budgets; and c) invest in mental health to address how fear and anxiety related to the pandemic intersects with pre-existing forms of stigmatization.

## Data availability

All data underlying the results are available as part of the article and no additional source data are required.

## Consent

Written informed consent for publication of the participants’ images was obtained from the participants.

## Footnotes

^i^ FIDA, 2008. Documenting Human Rights Violations of Sex Workers in Kenya A study conducted in Nairobi, Kisumu, Busia, Nanyuki, Mombasa and Malindi. Kenya.
