# Supplementary material for: HIV testing amid COVID-19: community efforts to reach men who have sex with men in three Kenyan counties
Source: Gates Open Res. 2020 Oct 29;4:117. Originally published 2020 Jul 29. [Version 2] doi: 10.12688/gatesopenres.13152.2 (PMC7477340; doi:10.12688/gatesopenres.13152.2)
Supplement: Peer review history

# Peer review: Reviewer response for version 1

**DOI:** 10.21956/gatesopenres.14346.r29237

**Published:** 2020-09-07

**Authors:** Venkatesan Chakrapani

**Affiliations:** 1 Centre for Sexuality and Health Research and Policy (C-SHaRP), Chennai, Tamil Nadu, India

- This open letter (“HIV testing amid COVID-19…” by Odinga et al.) comprehensively documents the challenges faced by MSM in three Kenyan cities in accessing HIV testing services amid the COVID situation and the steps taken by MSM-led organisations to increase access to HIV testing.
- Regarding context setting: The letter states that consensual adult same-sex sexual relationship is criminal in Kenya, an important structural element that has been shown to decrease access to HIV services. The authors can also add whether sex work is criminalised as well (as some MSM may engage in sex work). It will be helpful for the readers if the authors can briefly comment on whether access to services is more challenging for particular subgroups of MSM (in relation to sexual identities, sexual roles or gender expression, and sex work status) to understand the local context and intersectionality. For example, whether MSM in sex work and those who have feminine gender expression were more likely to not get tested for HIV when they needed it?
- COVID-related insurance for organisation staff: I understand that even after the initiation of nation-wide curfew, the outreach workers and peer educators continued to meet MSM face-to-face in the field and clinics. Was any COVID-related health insurance provided to the staff? If not, can it be considered? (It has implications for reallocations of the budget and the authors can state their views on it).
- Regarding self-testing: It will be better if the authors can clarify what happened to persons tested positive (in clinic-based testing and self-testing). Especially, whether those who tested positive in self-testing were asked to go for a ‘confirmatory’ HIV testing? For those MSM who have regular partners, one option would be to provide self-test kits to MSM to deliver them to their regular partners. Was that strategy considered; and if so, what was the experience in implementing it?
- Condom use and HIV testing: If relevant data are available, a brief discussion on access to condoms and sexual behaviours can be added as HIV testing is especially required for those who are not consistently using condoms in the past three months.
- Recommendation on decriminalisation: Given the negative impact of the criminalisation of consensual adult same-sex relationships on access to services and mental health, in their list of recommendations, the authors can consider adding decriminalisation of consensual adult same-sex sexual relationships as a structural intervention, as recommended by UNAIDS.
- Overall, the open letter is informative, and its recommendations will be useful for programme managers and policymakers.

Does the article adequately reference differing views and opinions?

Yes

Are all factual statements correct, and are statements and arguments made adequately supported by citations?

Yes

Is the Open Letter written in accessible language?

Yes

Where applicable, are recommendations and next steps explained clearly for others to follow?

Yes

Is the rationale for the Open Letter provided in sufficient detail?

Yes

Reviewer Expertise:

Access to HIV prevention and treatment, Stigma and discrimination, Health disparities faced by MSM and transgender women

I confirm that I have read this submission and believe that I have an appropriate level of expertise to confirm that it is of an acceptable scientific standard.

# Response

**Published:** 2020-09-07

**Authors:** Parinita Bhattacharjee

**Affiliations:** University of Manitoba, Kenya

Response to the Reviewer, 7 ^th^ September 2020

- This open letter (“HIV testing amid COVID-19…” by Odinga et al.) comprehensively documents the challenges faced by MSM in three Kenyan cities in accessing HIV testing services amid the COVID situation and the steps taken by MSM-led organisations to increase access to HIV testing.

Thank you so much.

- Regarding context setting: The letter states that consensual adult same-sex sexual relationship is criminal in Kenya, an important structural element that has been shown to decrease access to HIV services. The authors can also add whether sex work is criminalised as well (as some MSM may engage in sex work). It will be helpful for the readers if the authors can briefly comment on whether access to services is more challenging for particular subgroups of MSM (in relation to sexual identities, sexual roles or gender expression, and sex work status) to understand the local context and intersectionality. For example, whether MSM in sex work and those who have feminine gender expression were more likely to not get tested for HIV when they needed it?

Thank you so much. MSM in Kenya do engage in sex work and sex work is partially or fully criminalized in Kenya: The Kenyan Penal Code and the Sexual Offences Act do not criminalise sex work per se. These codes criminalise the actions of third parties associated with sex work, rather than focusing on sex workers themselves. However, Municipal by-laws across the country do directly criminalise sex work through articles outlawing “loitering for the purpose of prostitution,” “importuning” for the purpose of prostitution and “indecent exposure.”[[i]]. We will include this aspect in the revised version.

 

- COVID-related insurance for organisation staff: I understand that even after the initiation of nation-wide curfew, the outreach workers and peer educators continued to meet MSM face-to-face in the field and clinics. Was any COVID-related health insurance provided to the staff? If not, can it be considered? (It has implications for reallocations of the budget and the authors can state their views on it).

Thank you for the suggestion. This is currently not provided and we will add this as a recommendation in the revised version.

 

- Regarding self-testing: It will be better if the authors can clarify what happened to persons tested positive (in clinic-based testing and self-testing). Especially, whether those who tested positive in self-testing were asked to go for a ‘confirmatory’ HIV testing? For those MSM who have regular partners, one option would be to provide self-test kits to MSM to deliver them to their regular partners. Was that strategy considered; and if so, what was the experience in implementing it?

Thank you for the suggestion. The three MSM CHO led programmes provide ART in their clinics and hence all those who are tested positive through clinic based rapid testing are linked to ART. Kenya has a policy of “test and treat” for all. Hence anyone who is tested positive are expected to be linked to ART after counselling and consent. Those who are provided with HIV Self Testing kits are followed up and encouraged to visit the clinic for confirmatory test and other services. If tested positive through confirmatory test in the clinic, they are linked with ART services. In the revised version of the manuscript we will add the linkage to ART information.

While there is anecdotal data that MSM who receive HIVST also use the kit with their regular partner. As a project we have not explored that aspect and have not monitored it.

 

- Condom use and HIV testing: If relevant data are available, a brief discussion on access to condoms and sexual behaviours can be added as HIV testing is especially required for those who are not consistently using condoms in the past three months.

Thanks for the suggestion. In the revised version we can add data and discussion on condom distribution. The national guidelines in Kenya recommends quarterly clinical checkup and HIv testing for key populations on a quarterly basis.

 

- Recommendation on decriminalisation: Given the negative impact of the criminalisation of consensual adult same-sex relationships on access to services and mental health, in their list of recommendations, the authors can consider adding decriminalisation of consensual adult same-sex sexual relationships as a structural intervention, as recommended by UNAIDS.

Thank you for the suggestion. We can consider this suggestion in the revised version of the manuscript.

 

- Overall, the open letter is informative, and its recommendations will be useful for programme managers and policymakers.

Thank you.

[i] FIDA, 2008. Documenting Human Rights Violations of Sex Workers in Kenya A study conducted in Nairobi, Kisumu, Busia, Nanyuki, Mombasa and Malindi. Kenya

# Peer review: Reviewer response for version 1

**DOI:** 10.21956/gatesopenres.14346.r29235

**Published:** 2020-08-25

**Authors:** Jerry Okal

**Affiliations:** 1 Population Council, Nairobi, Kenya

The paper is well written and covers an important topic on HIV testing amid COVID-19 and how CBOs are configuring their services to adjust to the emerging challenges in services provision. That said, I feel the authors should have organized the respective CBOs response in terms of the challenges, and innovations (both short-term and long-term innovations) for HIV testing. If available it would be interesting to not only look at trends in HIV testing but the full HIV treatment cascade data during pre-and post- COVID-19 period.

 Also, given that we are in August can the data be updated to look at the trends in testing and HIV treatment cascade data if possible? Finally, what are some recommendations that the authors can give to governments, program managers and CBOs providing critical services to key population groups such as MSM in Kenya and other similar settings?

Does the article adequately reference differing views and opinions?

Yes

Are all factual statements correct, and are statements and arguments made adequately supported by citations?

Yes

Is the Open Letter written in accessible language?

Yes

Where applicable, are recommendations and next steps explained clearly for others to follow?

Yes

Is the rationale for the Open Letter provided in sufficient detail?

Yes

Reviewer Expertise:

Public health (HIV, RH, Gender based violence, Key and priority populations)

I confirm that I have read this submission and believe that I have an appropriate level of expertise to confirm that it is of an acceptable scientific standard.

# Response

**Published:** 2020-09-04

**Authors:** Parinita Bhattacharjee

**Affiliations:** University of Manitoba, Kenya

**Response to reviewer, 4 ^th^ September 2020:**

1. The paper is well written and covers an important topic on HIV testing amid COVID-19 and how CBOs are configuring their services to adjust to the emerging challenges in services provision.

**Thank you so much.**

2. That said, I feel the authors should have organized the respective CBOs response in terms of the challenges, and innovations (both short-term and long-term innovations) for HIV testing. If available, it would be interesting to not only look at trends in HIV testing but the full HIV treatment cascade data during pre- and post-COVID-19 period.

**Thank you for the feedback. As the focus of the paper was on HIV testing during COVID 19, we provided data related to HIV testing only. Based on your comments we are now providing linkage to treatment data. Please see the table below.**

3. Also, given that we are in August can the data be updated to look at the trends in testing and HIV treatment cascade data if possible?

**The time lag between submission and publication sometimes make the data outdated. Please find attached data here updated till July 2020 including treatment linkage data.**

4. Finally, what are some recommendations that the authors can give to governments, program managers and CBOs providing critical services to key population groups such as MSM in Kenya and other similar settings?

**Thank you for the feedback.**

**Recommendations to the government and donors:**

**a) Provide clear guidance – it is important to provide clear guidance on changes in programming due to COVID 19 or any other crisis. Guidance from government or donor helps in reducing fears and anxiety among implementers and key populations and allows both to be safe and responsible.**

**b) Be flexible with budgets and targets – COVID 19 created various new needs for a HIV prevention programme among KP including safety equipment for implementers, food and nutrition for key populations. The government and donors have to be flexible to address these immediate emerging needs.**

**c) Focus on mental health – COVID 19 brought with it fear and anxiety but also social isolation and depression. We recommend that HIV prevention programmes with key populations should integrate interventions to improve mental health among key populations to address the needs when crises like COVID 19 happen.**

****

**Recommendations to the implementers and CBOs:**

**COVID 19 has created a crisis that the implementers or CBOs did not prepare for. The recommendations are:**

**a) Assess and understand the needs of the KP community during this crisis and respond to the same - During the initial months of COVID 19 (March – May 2020)        pandemic, the need of the MSM were not condoms and lubricants but nutrition as many of the MSM had lost their livelihood. The MSM led CBOs had to raise funds through donors and community to address this immediate need.**

**b) Develop innovative strategies to reach the population and provide services – The pandemic led to several changes including close of hotspots, restrictions in mobility and having face to face meeting. The MSM led CBOs had to innovate by taking on the virtual route, home delivery of commodities etc. as described in the article.**

**c) Address the fears and anxiety around the new pandemic among implementing team and the KP community. The MSM led CBOs had to do several training and run virtual campaigns to provide correct information about COVID 19 and continuously remind people about safe behaviours (handwashing, wearing masks etc).**
